# Familial pancreatic cancer with *PALB2* and *NBN* pathogenic variants: a case report

**DOI:** 10.1186/s13053-020-00160-z

**Published:** 2021-01-07

**Authors:** Kodai Abe, Arisa Ueki, Yusaku Urakawa, Minoru Kitago, Tomoko Yoshihama, Yoshiko Nanki, Yuko Kitagawa, Daisuke Aoki, Kenjiro Kosaki, Akira Hirasawa

**Affiliations:** 1 Department of Surgery, Keio University School of Medicine, Tokyo, Japan; 2 Center for Medical Genetics, Keio University School of Medicine, Tokyo, Japan; 3 Department of Clinical Genomic Medicine, Graduate School of Medicine, Dentistry and Pharmaceutical Sciences, Okayama University, 2-5-1 Shikata-cho, Kita-ku, Okayama, 700-8558 Japan; 4 Department of Obstetrics and Gynecology, Keio University School of Medicine, Tokyo, Japan

**Keywords:** Hereditary pancreatic cancer, *PALB2*, *NBN*

## Abstract

**Background:**

Family history is one of the risk factors for pancreatic cancer. It is suggested that patients with pancreatic cancer who have a familial history harbor germline pathogenic variants of *BRCA1* and/or *BRCA2* (*BRCA1/2*), *PALB2*, or *ATM*. Recently, some germline variants of familial pancreatic cancers (FPCs), including *PALB2,* have been detected. Several countries, including Japan, perform screening workups and genetic analysis for pancreatic cancers. We have been carrying out active surveillance for FPC through epidemiological surveys, imaging analyses, and genetic analysis.

**Case presentation:**

Here, we present the case of a female patient harboring pathogenic variants of *PALB2* and *NBN*, with a family history of multiple pancreatic cancer in her younger brother, her aunt, and her father. Moreover, her father harbored a *PALB2* pathogenic variant and her daughter harbored the same *NBN* pathogenic variant. Given the *PALB2* and *NBN* variants, we designed surveillance strategies for the pancreas, breast, and ovary.

**Conclusions:**

Further studies are required to develop strategies for managing FPCs to facilitate prompt diagnosis before their progression.

## Background

Pancreatic cancer is one of the most lethal malignant diseases, despite the development of medical technology, including surgical skills and anticancer drugs. Moreover, it is difficult to detect pancreatic cancer through clinical examination due to the absence of novel biomarkers. Numerous recent studies have reported that some pancreatic cancers have hereditary factors and germline variants, especially in DNA repair genes. This is similar to the mechanism of hereditary breast and ovarian cancer (HBOC) syndrome caused by the *BRCA1/2* germline variant. In the National Clinical Cancer Network (NCCN) guidelines, revised in 2020, familial pancreatic cancer (FPC) was included in the HBOC syndrome because FPC harbors *BRCA1/2* germline variants to some extent [1]. Furthermore, recent studies have reported that *BRCA1/2* pathogenic variants potentially contribute to FPC and that the frequency of *BRCA1/2*, *PALB2*, and *ATM* variants in all pancreatic cancers is 7% [2–4].

Here, we report a pedigree with a lineage of strongly suspected FPC, resulting from *PALB2* and *NBN* pathogenic variants. PALB2, a partner and localizer of *BRCA2*, is a Fanconi anemia group protein (FANCN), which permits stable intranuclear localization and accumulation of BRCA2 and can cause pancreatic, breast, and ovarian cancer [5]. To our knowledge, this is the first reported case of a Japanese pedigree harboring both *PALB2* and *NBN* variants. Furthermore, this study describes a literature review on the latest reports and management strategies related to FPC.

## Case presentation

**Fig. 1:**
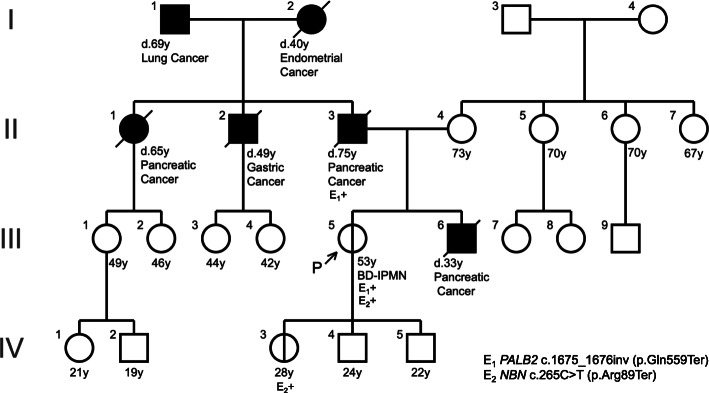
Family history of the pedigree. I-1: Dead from lung cancer at 69 years of age. I-2: Dead from endometrial cancer at 40 years of age. II-2: Dead from pancreatic cancer when she was 65 years of age. II-3: Dead from gastric cancer at 49 years of age. II-5: Diagnosed with unresectable pancreatic cancer after his son died. After the first genetic counseling, he underwent chemotherapy for cancer but died at 75 years of age. III-5: The *PALB2/NBN* pathogenic variants and branch-duct type IPMN. She has three children; a 28-year-old daughter (IV-3), a 24-year-old son (IV-4), and a 22-year-old son (IV-5). III-6: Diagnosed with advanced pancreatic adenocarcinoma at 32 years of age. He passed away despite undergoing chemotherapy for 10 months. IV-3: The *NBN* pathogenic variant

A 49-year-old woman at first consultation presented at our hospital for surveillance of the pancreas because her father (II-3) and her younger brother (III-6) had pancreatic cancer. She had undergone surgery for subarachnoid hemorrhage at 19 years of age because of an arteriovenous malformation. Her family tree (Fig. 1) revealed that her younger brother died of pancreatic cancer at 33 years of age; he could not be treated through surgery because of his advanced stage with distant metastasis. The patient’s paternal aunt (II-1) also died of pancreatic cancer at 65 years of age. Her father was also diagnosed with advanced-stage pancreatic cancer, which could not be controlled despite chemotherapy.

In the first genetic counseling session, the patient was informed that she was likely to have FPC, Lynch syndrome, or HBOC syndrome, all of which follow an autosomal dominant inheritance pattern. Therefore, germline multi-gene panel testing using ACTRisk® (ACT Genomics, Co. Ltd. Taipei, Taiwan) was performed to analyze germline variants in this case.

In the second genetic counseling session, we informed her that the blood genetic test revealed two germline variants. She harbored a heterozygous *PALB2* pathogenic variant, NM_024675(*PALB2*): c.1675_1676inv (p.Gln559*), and a heterozygous *NBN* pathogenic variant, NM_002485(*NBN*): c.265C > T (p.Arg89*). These variants are predicted to cause loss of normal protein function through either protein truncation or nonsense-mediated mRNA decay. Therefore, we advised her to undergo surveillance for breast, ovarian, and pancreatic cancer. Her father died 9 months after her first consultation; however, he had previously provided a blood sample to our department before his death to support her future healthcare. Accordingly, genetic testing of her father’s blood sample was recommended to her.

In the third genetic counseling session, we explained that her father’s blood revealed the presence of the *PALB2* c.1675_1676inv (p.Gln559) pathogenic variant, which was the same as hers. Furthermore, we informed her that her first-degree relatives (FDR) have a 50% chance of testing positive for these variants. Therefore, we recommended genetic counseling for her children at the next session, and she agreed.

In the fourth genetic counseling session, the patient and her three children, a 28-year-old woman, a 24-year-old man, and a 22-year-old man, presented at our outpatient department. We explained to them that their mother and her father harbored the *PALB2* pathogenic variant, which was probably associated with breast, ovarian, pancreatic, and prostate cancer. Furthermore, we informed them that their mother harbored the *NBN* pathogenic variant, which was potentially associated with breast, ovarian, and pancreatic cancer. Upon surveillance, no issue was noted in the cases’s breasts and ovaries; however, she displayed a branch duct type intraductal papillary mucinous neoplasm (BD-IPMN) in her pancreas. We suggested she continue active surveillance of her breasts, ovaries, and pancreas. Furthermore, her 28-year-old daughter wished to undergo genetic testing because her uncle had died from pancreatic cancer at an early age. Therefore, we performed genetic testing at a single site for the patient’s daughter. Finally, the patient’s daughter underwent genetic counseling and was found to harbor only the *NBN* c.265C > T(p.Arg89*) pathogenic variant, which was probably associated with breast, ovarian, pancreatic cancer. Thus, the daughter will be recommended to undergo surveillance for breast, ovarian, and pancreatic cancer.

## Discussion and conclusions

We reported a pedigree with FPC, harboring a *PALB2* pathogenic variant. Based on family history, the patient’s father and younger brother and her father’s sister had pancreatic cancer, whereas her mother’s family did not have a cancer history. Thus, her *PALB2* variant was inherited from her father, as revealed through genetic analysis. Furthermore, she had BD-IPMN, which can develop into pancreatic cancer. BD-IPMN cases are reportedly at a lower risk of pancreatic cancer than those with main-duct-type IPMN or mixed-type IPMN; however, invasive pancreatic cancer has reportedly occurred in approximately 3.8% of BD-IPMN cases after 84 months follow-up [6]. Hence, we intend to follow the present case annually using magnetic resonance imaging (MRI) or endoscopic ultrasound (EUS) imaging. Furthermore, the patient and her daughter harbored the *NBN* pathogenic variant, which reportedly elevates the risk of breast, ovarian, and pancreatic cancer.

A case of FPC was first reported in 1976 in a patient presenting multiple instances of pancreatic cancer in their family history [7]. The FPC registry was first developed at Johns Hopkins University in the United States in 1994. The Japanese Familial Pancreatic Cancer Registry (JFPCR) was established in 2014; it serves to recruit patients with pancreatic tumors and their first- and second-degree relatives. Furthermore, the JFPCR provides information regarding a patient’s family history and lifestyle [8, 9] but does not include germline variants [8, 9]. Germline variants of FPC have been reported at various institutes, and the most common germline variant is the DNA repair gene family, including *BRCA1/2*, *ATM*, *PALB2*, *CHEK2*, and *RAD51* [10].

The *BRCA2* variant is more frequent (approximately 4–17%) among patients with FPC than is *BRCA1*, and the *PALB2* variant has been observed in 2–3% of cases [11]. PALB2, which encodes a BRCA2-interacting protein, was first reported as a breast cancer susceptibility gene in 2007 [12]. Biallelic *PALB2* variants are responsible for a subset of Fanconi anemia cases characterized by a phenotype similar to that caused by biallelic *BRCA1/2* variants [13]. Most patients with FPC and HBOC syndrome harbor monoallelic *PALB2* or *BRCA1/2* variants, which follow an autosomal dominant inheritance pattern [13].

*NBN*, discovered as a cause of Nijmegan breakage syndrome through biallelic pathogenic variants, is a type of DNA damage response gene that is associated with severe microcephaly, infertility, and cancer risk [14]. The 95-kDa NBN protein has 754 amino acid residues and is a component of the MRN complex comprising *MRE11*, *RAD50*, and *NBN*. The MRN complex is one of the first players to detect double-stranded breaks and be recruited to chromatin through interactions between poly-ADP-ribose (PAR) and its first BRCT domain [15]. *NBN* is potentially susceptible to variation in breast and ovarian cancers [16]; however, this pathogenic variant is less known than *BRCA1/2*, *ATM*, or *PALB2*. Moreover, recent studies have reported that the *NBN* variant is associated with pancreatic cancer [17, 18]. In our case, the *NBN* variant may have been inherited from her mother’s lineage or may have originated de novo, although no studies have reported de novo *NBN* variants. The patient’s daughter harbored the same *NBN* variant; hence, continued surveillance of the breast, ovary, and pancreas is needed.

One of the most important considerations from this case is determining how we should perform cancer surveillance for unaffected family members who harbor germline variants. HBOC is the most frequent hereditary tumor, and management guidelines have been established worldwide; however, no standardized surveillance methods are available for FPC. Furthermore, no guidelines for non-*BRCA1/2* HBOC are available in Japan, despite the increasing number of non-*BRCA1/2* HBOC cases. To resolve these issues, surveillance for FPC and management guidelines for HBOC and FPC should be considered as soon as possible.

According to the report from the evidence-based network for the interpretation of germline mutant alleles (ENGIMA) clinical working group, breast cancer surveillance for *PALB2* is different across countries [19]. For example, the United States, France, Spain, Australia, and Germany recommend an annual mammogram and breast MRI from the age of 30 years and consider reduced risk mastectomy (RRM), whereas Belgium, the United Kingdom, and Japan have no guidelines regarding *PALB2* management. The guideline regarding *NBN* surveillance in the United States is for individuals with the *NBN* variant to undergo mammograms and breast MRIs from the age of 40 years. The Japanese management of non-*BRCA1/2* pathogenic variants, including *PALB2*, *ATM*, and *NBN,* should be concreated because non-*BRCA1/2* genes are greatly associated with hereditary pancreatic cancer.

Our institute performs pancreatic cancer surveillance for cases with germline variants in accordance with the International Cancer of Pancreas Screening (CAPS) established in 2013 by Johns Hopkins University [20]. We denote the following as high-risk individuals (HRI): persons with three or more blood relatives with pancreatic cancer, those with at least two affected FDRs, patients with Peutz-Jeghers syndrome, and those who already harbor germline variants in *BRCA1/2*, *PALB2*, *ATM*, *CDKN2A*, *MLH1*, *MSH2*, *MSH6*, *EPCAM*, *STK11*, and *TP53*. We then perform abdominal MRIs for HRI and, if they have no issue in the pancreas, we recommend an annual abdominal MRI. If they have issues in the pancreas, we perform endoscopic ultrasound (EUS). This surveillance has been recommended in accordance with the NCCN guidelines 2020 Ver1 [1]. Accordingly, the patient and her daughter in this case study need to undergo breast, ovary, and pancreatic cancer surveillance, and the her two sons need to undergo genetic counseling.

In conclusion, we reported a family with hereditary pancreatic cancer harboring pathogenic variants of both *PALB2* and *NBN.* The *PALB2* pathogenic variant in our case was inherited from her father, and her *NBN* pathogenic variant was inherited by her daughter. This case indicated that we should share and generalize our knowledge and perform active surveillance of HRIs for pancreatic cancer. Furthermore, a prospective cohort study and registration system, including gene variants to establish national or international guidelines, is required. In conclusion, an increased number of studies on FPC would help establish management guidelines for the surveillance or screening of pancreatic cancer to detect early-stage cancer and improve prognosis.

## Abbreviations

HBOC: Hereditary breast and ovarian cancer
NCCN: National Clinical Cancer Network
FPC: Familial pancreatic cancer
FANCN: Fanconi anemia group protein
DSB: Double-strand break
FDR: First-degree relative
EUS: Endoscopic ultrasound
JFPCR: Japanese Familial Pancreatic Cancer Registry

## References

[1] NCCN (2019). Clinical Practice Guidelines in Oncology (NCCN Guidelines®). Genetic/Familial High-Risk Assessment: Breast, Ovarian, and Pancreatic. Version 1.2020.

[2] Takai E, Yachida S, Shimizu K, Furuse J, Kubo E, Ohmoto A (2016). Germline mutations in Japanese familial pancreatic cancer patients. Oncotarget.

[3] Pittman ME, Brosens LAA, Wood LD (2016). Genetic syndromes with pancreatic manifestations. Surg Pathol.

[4] Waddell N, Pajic M, Patch A, Chang DK, Kassahn KS, Bailey P (2015). Whole genomes redefine the mutational landscape of pancreatic cancer. Nature.

[5] Nepomuceno MC, Gregoriis GD, Bastos de Oliveira FM, Suarez-Kurtz G, Monteiro AN, Carvalho MA (2017). The role of PALB2 in the DNA damage response and cancer predisposition. Int J Mol Sci.

[6] Khannoussi W, Vullierme M, Rebours V, Maire F, Hentic O, Aubert A (2012). The long-term risk of malignancy in patients with branch duct intraductal papillary mucinous neoplasms of the pancreas. Pancreatology.

[7] Friedman JM, Fialkow PJ (1976). Familial carcinoma of the pancreas. Clin Genet.

[8] Morizane C, Kitano M, Hijioka S, Ito T, Kamisawa T, Kosugi S (2017). The familial pancreatic cancer registration for early detection. Suizo.

[9] Japan Familial Pancreatic Cancer Registration (JFPCR). http://jfpcr.com/. Accessed 4 Apr 2020.

[10] Rustgi AK (2014). Familial pancreatic cancer: genetic advances. Genes Dev.

[11] Roberts NJ, Norris AL, Petersen GM, Bondy ML, Brand R, Gallinger S (2016). Whole genome sequencing defines the genetic heterogeneity of familial pancreatic cancer. Cancer Discov.

[12] Rahman N, Seal S, Thompson D, Kelly P, Renwick A, Elliott A (2007). PALB2, which encodes a BRCA2- interacting protein, is a breast cancer susceptibility gene. Nat Genet.

[13] Ramus SJ, Song H, Dicks E, Yrer JP, Rosenthal AN, Intermaggio MP (2015). Germline mutations in the BRIP1, BARD1, PALB2, and NBN genes in women with ovarian cancer. J Natl Cancer Inst.

[14] Gałązka P, Czyżewski K, Szaflarska-Popławska A, Dębski R, Krenska A, Styczyński J (2019). Complex profile of multiple hepatobiliary and gastrointestinal complications after hematopoietic stem cell transplantation in a child with Nijmegen breakage syndrome. Cent Eur J Immunol.

[15] Fiévet A, Bellanger D, Zahed L, Burglen L, Derrien AC, Dubois d’Enghien C (2019). DNA repair functional analyses of NBN hypomorphic variants associated with NBN-related infertility. Hum Mutat.

[16] Gass J, Jackson J, Macklin S, Blackburn P, Hines S, Atwal PS (2017). A case of contralateral breast cancer and skin cancer associated with NBN heterozygous pathogenic variant c.698_701delAACA. Fam Cancer.

[17] Borecka M, Zemankova P, Lhota F, Soukupova J, Kleiblova P, Vocka M (2016). The c.657del5 variant in the NBN gene predisposes to pancreatic cancer. Gene.

[18] Lener MR, Scott RJ, Kluzniak W, Baszuk P, Cybulski C, Wiechowska-Kozłowska A (2016). Do founder mutations characteristic of some cancer sites also predispose to pancreatic cancer?. Int J Cancer.

[19] Nielsen SM, Eccles DM, Romero IL, Al-Mulla F, Balmaña J, Biancolella M (2018). Genetic testing and clinical management practices for variants in non-BRCA1/2 breast (and breast/ovarian) cancer susceptibility genes: an international survey by the evidence-based network for the interpretation of Germline mutant alleles (ENIGMA) clinical working group. JCO Precis Oncol.

[20] Canto MI (2013). International Cancer of the pancreas screening (CAPS) consortium summit on the management of patients with increased risk for familial pancreatic cancer. Gut.

